# Supplementary material for: Prediction of 3D Cardiovascular hemodynamics before and after coronary artery bypass surgery via deep learning
Source: Commun Biol. 2021 Jan 22;4:99. doi: 10.1038/s42003-020-01638-1 (PMC7822810; doi:10.1038/s42003-020-01638-1)
Supplement: Supplementary file 2 — Supplementary Information [file 42003_2020_1638_MOESM2_ESM.pdf]

Prediction of 3D Cardiovascular Hemodynamics before and after Coronary Artery Bypass Surgery via Deep Learning

Gaoyang Li et al.

Supplementary Information

Supplementary Results

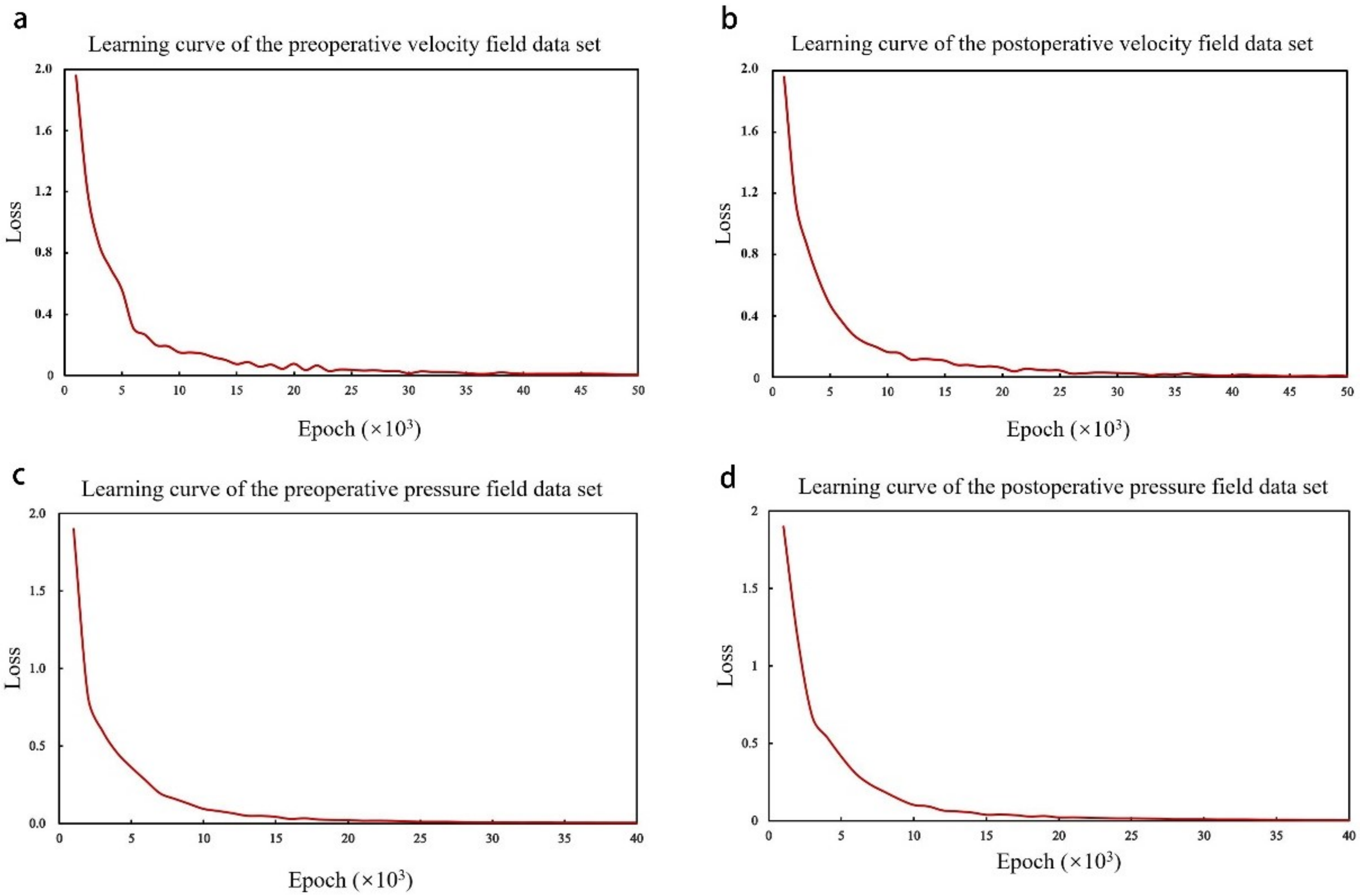

Supplementary Fig. 1 Learning curve. **a-b** are the learning curves of the pre- and post-velocity field datasets. **c-d** are the learning curves of the pre- and post-pressure field datasets. For velocity, the learning curve reaches a minimum when Epoch is about 50K. For pressure, the learning curve reaches a minimum when Epoch is about 40K.

Supplementary Discussion

Comparison against Previous Deep Learning Approaches

We reviewed studies using deep learning or machine learning for prediction of flow fields or clinical parameters related to CHD treatment (e.g., FFR), as shown in Supplementary Table 1. Itu's machine learning method was only suitable for the calculation of FFR value[1], and its application range was extremely limited. Lee's convolutional neural network could be used for vortex flow prediction in the 2D plane[2], which might lead to loss of information (such as the flow field component perpendicular to the 2D plane). Guo's deconvolution network is only suitable for 3D regular and simple flow field[3]. Because the network structure is relatively simple, the above studies all need a large number of data samples to support in order to achieve high prediction accuracy. Although Liang realizes the internal hemodynamic prediction of the ideal thoracic aortic model[4], the spatial resolution of its samples is still low, which could not accurately characterize the geometric characteristics of complex cardiovascular system. Liang's network only accepts the input data with prefix array-size. This means, patient geometry should be normalized into template (fixed number of meshes). Then, if the geometry cannot fit into that template, Liang's network cannot accept that input. Under the premise of more extensive information, our deep learning method uses limited data to achieve prediction accuracy similar to previous studies. However, our prediction objects are far more complex. Our network can predict the flow on any kind of geometry owing to using the point cloud format. Even the number of point cloud (nodes) varies, our network can accept that unfixed input. Combined with the universality analysis of the network, our deep learning method has many advantages.

Supplementary Table 1 Comparison analysis of our deep learning method against previous studies

Network or method	Prediction output	Data set size	Input data format	Error function or accuracy
Our Deep Learning Method	3D Patient Personalized Cardiovascular Hemodynamics	1100	High resolution flexible point cloud	NMAE<6.5%, MRE<10%
Itu's Machine Learning Approach[1]	FFR Value	12,000	Geometric parameters	Accuracy= 99.7%
Lee's Adversarial and Convolutional Neural Networks[2]	2D Vortex Flow	500000	Grid cells with fixed number	32.8%<Error<1%
Guo's Deconvolution Network[3]	3D Regular and Simple Flow	400000	Low resolution pixels with fixed number	MRE<3%
Liang's DNNs[4]	3D Ideal Thoracic Aorta Hemodynamics	729	Low resolution meshes with fixed number	NMAE<6.5%

Supplementary Method

Model geometric parameters modification

In order to visually show the difference between the models in the training set and the test set, and to clearly show the modification of the model's geometric structure, we selected a model from the training set and the test set, and showed the modification results of its geometric structure, as shown in Supplementary Fig. 2.

Training set

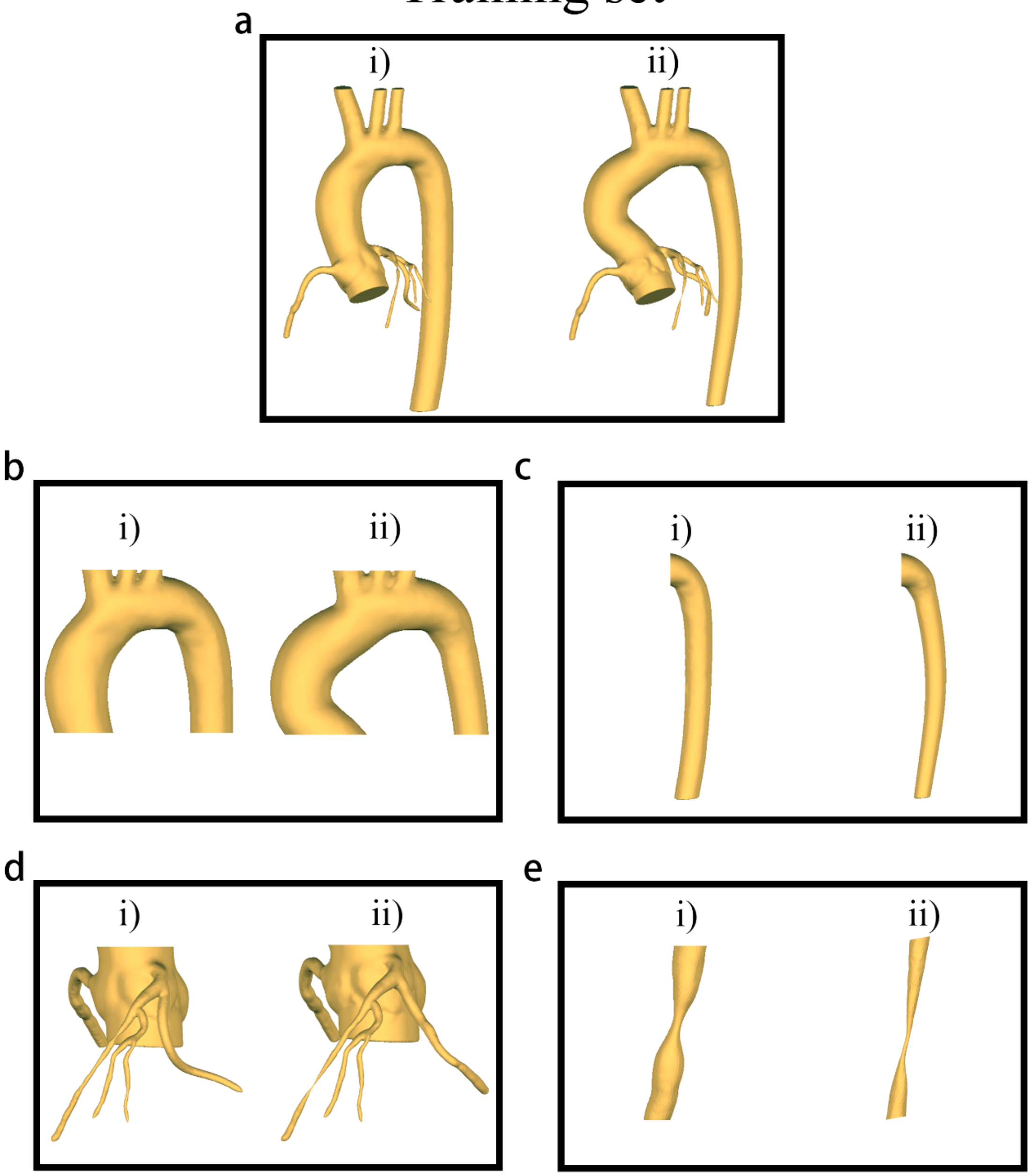

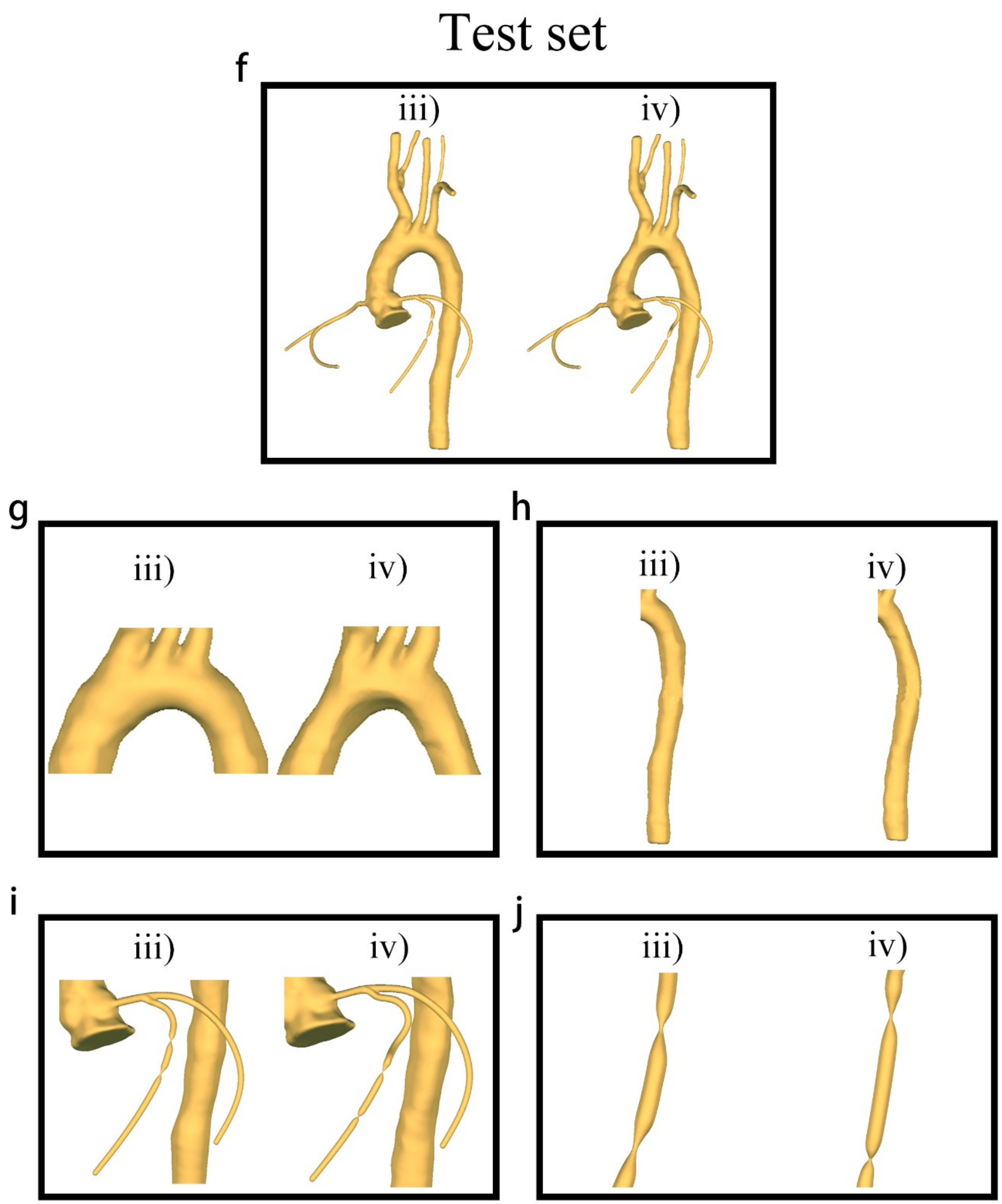

Supplementary Fig. 2 Examples of models in the training and test sets. Examples of models in the training and test sets. i) and iii) is the original model. ii) and iv) is the corresponding modified model. **a** and **f**: overall model; **b** and **g**: ascending aorta and aortic arch angulation; **c** and **h**:

descending aorta; **d** and **i**: coronary artery details (LAD and LCX); **e** and **j**: stenosis. All model modifications follow the provisions of Table 3 in the main text.

Simulated operation of CABG and CFD simulation

Among these patient-specific models, except for a few patients who had undergone CABG surgery, the vast majority of patient data had not undergone CABG surgery. Therefore, virtual CABG bypass surgery was performed on these models. According to the recommendations of existing patients and doctors, we chose the left internal mammary artery (LIMA) as the bypass graft. The diameter of the blood vessel was 2 mm following the advice of the doctors. In Supplementary Fig. 3, we chose one of the models as an example to show before and after CABG surgery. The operation of virtual surgery was done using the commercially available software Mimics (Materialize NV, BE). Before generating the computational models, the reconstructed 3D models needed to be preprocessed, including surface smoothing and inlet/outlets processing by using the commercially available software Geomagic Wrap (3D system, US). After model preprocessing, tetrahedron-dominant mesh computational models were generated, with maximal sizes of 1.6 mm for the element, for each patient model before and after the CABG procedure using ANSYS-Meshing (ANSYS, Canonsburg, USA). To better capture the flow behaviors, close to the vascular wall, five prismatic boundary layers were generated with a growing ratio of the prism thickness at 1.2 mm[5-8].

The vascular wall was assumed to be rigid and a non-slip condition was assigned at all boundaries. We assumed the blood to be an incompressible Newtonian fluid, with density and viscosity of 1050 kg/m^3 and 0.0035 Pa s, respectively, and performed steady flow simulations using solver ANSYS-CFX (ANSYS, Canonsburg, USA). Since the FFR calculation needed to be in the state of maximum congestion, a peak wave velocity of 1.125 m/s was imposed as the boundary condition at the inlet[9]. And for the outlets, a fixed static reference zero pressure was applied. The

convergence criteria for simulations were chosen as 10^{-4} (Root Mean Square) for the normalized continuity, pressure, and velocity residuals. Upon convergence of each simulation, we extracted four groups of results including preoperative velocity fields and pressure distributions and postoperative velocity fields and pressure distributions and saved them as a CSV file, for further processing and construction of the dataset to be used in deep learning.

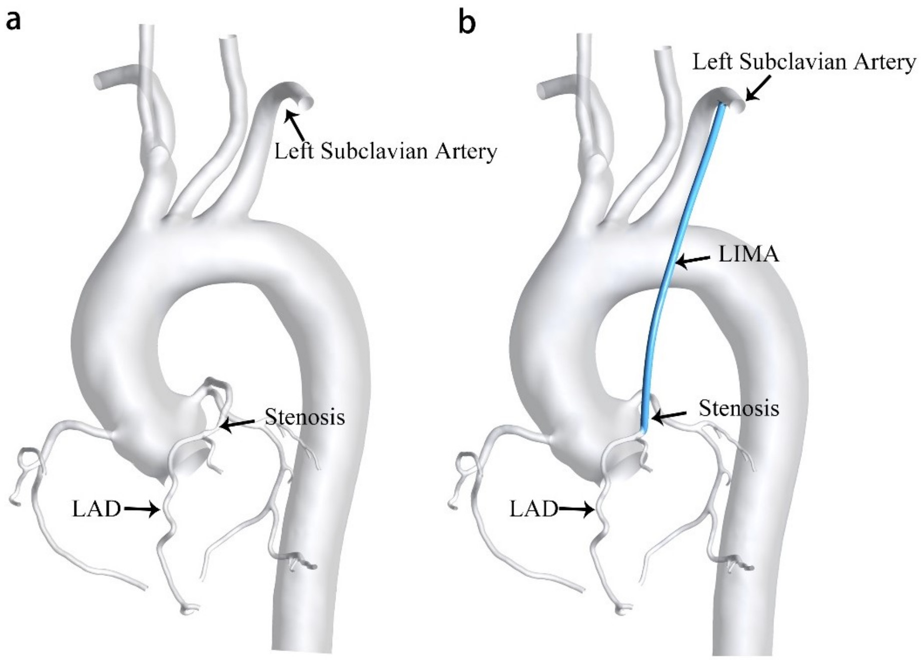

Supplementary Fig. 3 Simulated operation of CABG.

Mesh Independence Test

In this study, the number of nodes ranged from 2.83 to 3.01 million in total across different cases. In order to reduce errors and make the simulation results stable and reliable, a mesh independence test of computational grids was performed. As shown in Supplementary Fig. 4, the relationship between the mass-flow of LIMA and the number of grids. When the number of meshes exceeds one million, the calculation results can be considered to be stable. Therefore, it is confirmed that the number of grids set in this study was appropriate.

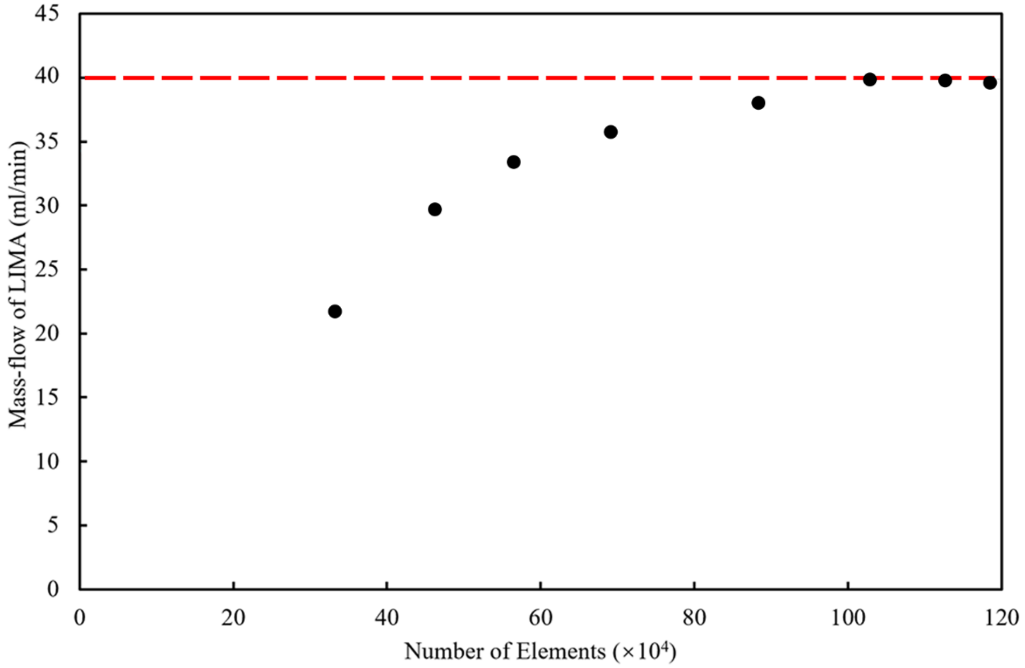

Supplementary Fig. 4 Mesh independence test. It confirms that the mesh setting of this study is suitable.

Supplementary References

1. Itu, L. *et al.* A machine-learning approach for computation of fractional flow reserve from coronary computed tomography. *J. Appl. Physiol.* **121**, 42–52 (2016).

2. Lee, S. & You, D. Prediction of laminar vortex shedding over a cylinder using deep learning. *arXiv Prepr. arXiv1712.07854* (2017).

3. Guo, X., Li, W. & Iorio, F. Convolutional neural networks for steady flow approximation. in *Proceedings of the 22nd ACM SIGKDD international conference on knowledge discovery and data mining* 481–490 (2016).

4. Liang, L., Mao, W. & Sun, W. A feasibility study of deep learning for predicting hemodynamics of human thoracic aorta. *J. Biomech.* **99**, 109544 (2020).

5. Taylor, C. A., Hughes, T. J. R. & Zarins, C. K. Finite element modeling of blood flow in arteries. *Comput. Methods Appl. Mech. Eng.* **158**, 155–196 (1998).

6. Longest], P. [Worth & Kleinstreuer, C. Comparison of blood particle deposition models for non-parallel flow domains. *J. Biomech.* **36**, 421–430 (2003).

7. Chiou, M. C. Particle deposition from natural convection boundary layer flow onto an isothermal vertical cylinder. *Acta Mech.* **129**, 163–176 (1998).

8. Dyedov, V. *et al.* Variational Generation of Prismatic Boundary-Layer Meshes for Biomedical Computing. *Int. J. Numer. Methods Eng.* **79**, 907–945 (2009).

9. Febina, J., Sikkandar, M. Y. & Sudharsan, N. M. Wall Shear Stress Estimation of Thoracic Aortic Aneurysm Using Computational Fluid Dynamics. *Comput. Math. Methods Med.* https://doi.org/10.1155/2018/7126532 (2018)
