# Supplementary material for: Prediction of 3D Cardiovascular hemodynamics before and after coronary artery bypass surgery via deep learning
Source: Commun Biol. 2021 Jan 22;4:99. doi: 10.1038/s42003-020-01638-1 (PMC7822810; doi:10.1038/s42003-020-01638-1)
Supplement: Supplementary file 3 — Reporting Summary [file 42003_2020_1638_MOESM3_ESM.pdf]

nature research

Corresponding author(s): Hitomi Anzai

Last updated by author(s): Nov 27, 2020

Reporting Summary

Nature Research wishes to improve the reproducibility of the work that we publish. This form provides structure for consistency and transparency in reporting. For further information on Nature Research policies, see our Editorial Policies and the Editorial Policy Checklist.

Statistics

For all statistical analyses, confirm that the following items are present in the figure legend, table legend, main text, or Methods section.

n/a	Confirmed	
☐	☒	The exact sample size (*n*) for each experimental group/condition, given as a discrete number and unit of measurement
☐	☒	A statement on whether measurements were taken from distinct samples or whether the same sample was measured repeatedly
☐	☒	The statistical test(s) used AND whether they are one- or two-sided<br>*Only common tests should be described solely by name; describe more complex techniques in the Methods section.*
☒	☐	A description of all covariates tested
☐	☒	A description of any assumptions or corrections, such as tests of normality and adjustment for multiple comparisons
☐	☒	A full description of the statistical parameters including central tendency (e.g. means) or other basic estimates (e.g. regression coefficient) AND variation (e.g. standard deviation) or associated estimates of uncertainty (e.g. confidence intervals)
☐	☒	For null hypothesis testing, the test statistic (e.g. *F*, *t*, *r*) with confidence intervals, effect sizes, degrees of freedom and *P* value noted<br>*Give P values as exact values whenever suitable.*
☒	☐	For Bayesian analysis, information on the choice of priors and Markov chain Monte Carlo settings
☐	☒	For hierarchical and complex designs, identification of the appropriate level for tests and full reporting of outcomes
☒	☐	Estimates of effect sizes (e.g. Cohen's *d*, Pearson's *r*), indicating how they were calculated

Our web collection on statistics for biologists contains articles on many of the points above.

Software and code

Policy information about availability of computer code

Data collection	The patient data used in this study was based on the project 'Biomechanics study on quantitative relationships between coronary artery stenosis and myocardial ischemia', which focused on the diagnosis and optimization of coronary stenosis surgical procedures. The CTA data for 110 patients with LAD stenosis who had visited the People's hospital since 2018 was collected and collated by professional clinicians with a 128-slice CT scanner (Brilliance iCT, Philips Healthcare, The Netherlands). 3D model reconstruction was also performed by the clinicians. We obtained 110 STL cardiovascular models as raw data.
Data analysis	The operation of virtual surgery was done using the commercially available software Mimics v17.0 (Materialize NV, BE). Before generating the computational models, the reconstructed 3D models needed to be preprocessed, including surface smoothing and inlet/outlets processing by using the commercially available software Geomagic Wrap (3D system, US). After model preprocessing, tetrahedron-dominant mesh computational models were generated, with maximal sizes of 1.6 mm for the element, for each patient model before and after the CABG procedure using ANSYS-Meshing v19.2 (ANSYS, Canonsburg, USA). To better capture the flow behaviors, close to the vascular wall, five prismatic boundary layers were generated with a growing ratio of the prism thickness at 1.2 mm.<br>The vascular wall was assumed to be rigid and a non-slip condition was assigned at all boundaries. We assumed the blood to be an incompressible Newtonian fluid, with density and viscosity of 1050 kg/m3 and 0.0035 Pa s, respectively, and performed steady flow simulations using solver ANSYS-CFX v19.2 (ANSYS, Canonsburg, USA).<br>All source code described in this project can be accessed at: https://doi.org/10.5281/zenodo.4287103<br>The simulation software used is explained in detail in the article.

For manuscripts utilizing custom algorithms or software that are central to the research but not yet described in published literature, software must be made available to editors and reviewers. We strongly encourage code deposition in a community repository (e.g. GitHub). See the Nature Research guidelines for submitting code & software for further information.

Data

Policy information about availability of data

All manuscripts must include a data availability statement. This statement should provide the following information, where applicable:

- Accession codes, unique identifiers, or web links for publicly available datasets
- A list of figures that have associated raw data
- A description of any restrictions on data availability

Data analyzed during the current study are available from the corresponding author upon reasonable request. Restrictions apply to the sharing of patient data that supports the findings of this study. With the approval of the Institutional Ethics Committee of People's Hospital, the patient's data can be authorized for use by qualified researchers.
The source data underlying the graphs and charts presented in the main figures (from Fig 1 to Fig 4) can be accessed at: https://doi.org/10.6084/m9.figshare.13295915.v1

Field-specific reporting

Please select the one below that is the best fit for your research. If you are not sure, read the appropriate sections before making your selection.

☒ Life sciences ☐ Behavioural & social sciences ☐ Ecological, evolutionary & environmental sciences

For a reference copy of the document with all sections, see nature.com/documents/nr-reporting-summary-flat.pdf

Life sciences study design

All studies must disclose on these points even when the disclosure is negative.

Sample size	The CTA data for 110 patients with LAD stenosis who had visited the People's hospital from 2018 to 2019 was collected and collated by professional clinicians with a 128-slice CT scanner (Brilliance iCT, Philips Healthcare, The Netherlands). 3D model reconstruction was also performed by the clinician. We obtained 110 STL cardiovascular models as raw data.<br>The deep learning dataset, which only contained 110 real cardiovascular models, had a very limited amount of information, which was far from enough to represent the relationship between the geometry of the model and the corresponding hemodynamics. Therefore, based on the statistical results of previous cardiovascular morphology studies, the geometric parameters of the 110 original cardiovascular models were adjusted to increase the number of models. For each parameter, we randomly selected one value within the given range as the modification basis of the original model, as shown in Table 3. Based on this method, we extended one original model into nine new models, which meant that the total number of models increased to 1100.<br>We reviewed studies using deep learning or machine learning for prediction of flow fields or clinical parameters related to CHD treatment (e.g., FFR), as shown in Supplementary Table 1. Under the premise of more extensive information, our deep learning method uses limited data to achieve prediction accuracy similar to previous studies. However, our prediction objects are far more complex. It can prove that the amount of data in this study is sufficient.
Data exclusions	For the purpose of this study, the exclusion criteria of the original data are as follows under the advice of professional doctors:<br>1. Exclude the CTA image with poor clarity and quality.<br>2. Patients with obvious cardiovascular distortion were excluded.<br>3. Patients with non LAD stenosis were excluded.
Replication	For the deep learning training set and test set, we use random assignment to ensure the generalization of the network and the repeatability of the experimental results.
Randomization	For deep learning, the distribution of samples in training set and test set is random
Blinding	This study does not include the comparative analysis of deep learning prediction results and clinical medical data. Therefore, blinding is not relevant to our study.

Reporting for specific materials, systems and methods

We require information from authors about some types of materials, experimental systems and methods used in many studies. Here, indicate whether each material, system or method listed is relevant to your study. If you are not sure if a list item applies to your research, read the appropriate section before selecting a response.

Materials & experimental systems

n/a	Involved in the study
☒	☐ Antibodies
☒	☐ Eukaryotic cell lines
☒	☐ Palaeontology and archaeology
☒	☐ Animals and other organisms
☐	☒ Human research participants
☒	☐ Clinical data
☒	☐ Dual use research of concern

Methods

n/a	Involved in the study
☒	☐ ChIP-seq
☒	☐ Flow cytometry
☒	☐ MRI-based neuroimaging

Human research participants

Policy information about studies involving human research participants

Population characteristics

Based on the research purpose of this study, we do not care about the patient's age, gender and other characteristics. All samples in this study were patients with left anterior descending coronary stenosis, including some patients who underwent coronary bypass surgery.

Recruitment

The patient data used in this study was based on the project 'Biomechanics study on quantitative relationships between coronary artery stenosis and myocardial ischemia', which focused on the diagnosis and optimization of coronary stenosis surgical procedures. The CTA data for 110 patients with LAD stenosis who had visited the People's hospital since 2018 was collected and collated by professional clinicians with a 128-slice CT scanner (Brilliance iCT, Philips Healthcare, The Netherlands).
The data for this study comes from a project optimizing the treatment plan of coronary stenosis. Therefore, our datasets do not contain information on other cardiovascular diseases such as coronary aneurysms or aortic diseases. This results in our inability to verify the applicability of this study to other cardiovascular diseases. We give a detailed explanation in the discussion section.

Ethics oversight

The experimental scheme and related details of this study were approved by the Institutional Ethics Committee of People's Hospital (Beijing, China) and Tohoku University (Sendai, Miyagi, Japan). All experiments were carried out in accordance with relevant guidelines and regulations. We explained research content to the subjects in detail and obtained their written informed consent.

Note that full information on the approval of the study protocol must also be provided in the manuscript.
